# Threonine Requirements in Dietary Low Crude Protein for Laying Hens under High-Temperature Environmental Climate

**DOI:** 10.3390/ani9090586

**Published:** 2019-08-21

**Authors:** Mahmoud Mostafa Azzam, Rashed Alhotan, Abdulaziz Al-Abdullatif, Saud Al-Mufarrej, Mohammed Mabkhot, Ibrahim Abdullah Alhidary, Chuntian Zheng

**Affiliations:** 1 Animal Production Department, College of Food and Agriculture Sciences, King Saud University, Riyadh 11451, Saudi Arabia; 2 Guangdong Key Laboratory of Animal Breeding and Nutrition/Guangdong Public Laboratory of Animal Breeding and Nutrition/The Key Laboratory of Animal Nutrition and Feed Science (South China) of Ministry of Agriculture/State Key Laboratory of Livestock and Poultry Breeding/Institute of Animal Science, Guangdong Academy of Agricultural Sciences, Guangzhou 510640, China; 3 Poultry Production Department, Faculty of Agriculture, Mansoura University, Mansoura 35516, Egypt

**Keywords:** cholesterol, CuZn-SOD, HMG-CoA, HSP70, laying hens, L-Thr

## Abstract

**Simple Summary:**

The threonine (Thr) requirement of laying hens in a high-temperature climate is scarcely referred in the review of literature. Therefore, our aim was to estimate the dietary Thr requirement in low CP diets in a high-temperature environmental climate. Based on our findings, the optimal dietary Thr requirements to optimize egg production, serum uric acid, and serum CuZn-SOD were 0.58%, 0.59%, and 0.56%, respectively, by regression analysis.

**Abstract:**

Lohmann Brown hens (n = 420), at 28 weeks of age, were divided into five dietary treatments, and each treatment included six replicates of 14 laying hens. Dietary crude protein (14%) was presented as the control diet. Dietary L-Thr was added to the control diet for 12 weeks. Dietary Thr levels are 0.43%, 0.49%, 0.57%, 0.66%, and 0.74%, based on digestible base. From 28 to 40 weeks, hen-day egg production presented a quadratic trend to supplementing dietary Thr (R^2^ = 0.96, *p* = 0.02), and reached a maximum level at 0.58%. Serum uric acid demonstrated a quadratic trend (R^2^ = 0.62, *p* = 0.02) at 0.59%. Both serum total cholesterol and 3-hydroxy-3-methylglutaryl (HMG-CoA) reductase showed lower levels (*p* < 0.05) at 0.66% Thr. Serum CuZn-SOD elevated (*p* < 0.05) at 0.49%, 0.57%, and 0.66% Thr, as compared to the control group, and showed a quadratic trend (R^2^ = 0.87, *p* = 0.003) at 0.56%. Supplemental L-Thr decreased (*p* < 0.05) the expression of ileal HSP70 at 0.66% Thr. In summary, the optimal dietary Thr requirements to optimize egg production, serum uric acid, and serum CuZn-SOD were 0.58%, 0.59%, and 0.56%, respectively, by regression analysis.

## 1. Introduction

High temperatures negatively affect protein utilization efficiency [1]. In addition, diets that contain a high content of dietary crude protein (CP) will increase internal heat production by the elevated heat increment in a high-temperature climate [2]. Therefore, low levels of CP with supplementing limiting amino acids can overcome the bad effects of heat stress [3,4] and enhance protein utilization [5,6]. Recently, it has been reported that laying performance was equal among 14%, 15%, and 16% dietary CP [7]. Since synthetic dietary L-Thr became commercially available, it is possible to decrease CP. Thr affects protein synthesis and is the third limiting amino acid [8,9]. Recently, it has been reported that Thr is a limiting amino acid in diets containing 14% CP [10].

It has been showed that heat stress provoked lipid accumulation by elevated de novo lipogenesis, decreased lipolysis, and enhanced amino acid catabolism [11]. In addition, during stress times, the bird’s body begins freeing heat shock proteins to secure itself from the harmful cellular effects of reactive oxygen species [12]. Heat stress is usually accompanied by increasing levels of 70 kilodalton heat shock proteins (HSP70) [13,14]. In addition, high temperatures disturb oxidative status [15] and increase serum total cholesterol, triglyceride, and zinc [16,17,18,19,20].

The present research aimed to estimate the dietary Thr requirement in low CP diets for laying hens in a high-temperature environmental climate. In addition, the effects of increasing Thr on lipid peroxidation, antioxidants enzymes activities, mineral levels, and HSP70 were investigated.

## 2. Material and Methods

### 2.1. Management

All procedures in this study were conducted according to the guide for the care and use of agricultural animals in research and teaching (American society of animal science and poultry science association, 2010), through research group (No. RG-1440-146).

**Figure 1:**
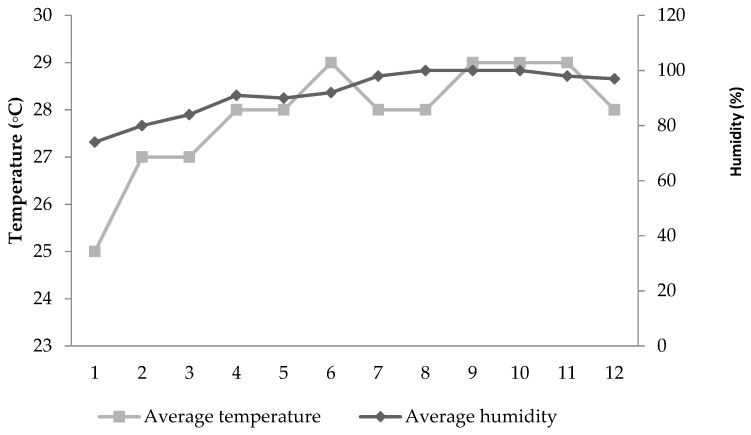
Average temperature (°C) and humidity (%) inside the experimental farm by week.

Lohmann Brown hens (n = 420), at 28 weeks of age, and with almost similar live body weights (1800 g), were divided into five dietary treatments. Each treatment included six replicates of 14 laying hens (4 birds/cage; 471.5 cm^2^/hen). They were exposed to 16-h light. The study began during the middle of May and ended in August; it lasted 13 weeks, including one week for acclimation. The mean daily temperature and humidity are both presented in Figure 1.

### 2.2. Experimental Diets

Hens were fed *adlibitum* (mash form), and water was available through nipples. Dietary CP (14%) was presented as the control diet (Table 1). Dietary Thr levels are 0.43%, 0.49%, 0.57%, 0.66%, and 0.74%, based on digestible base. Ingredient and analyzed CP and total amino acids are presented in Table 1 and Table 2, respectively. L-Thr (98.5% purity) was supplied at the expense of kaolin (inert filler). Samples from each diet were analyzed for CP and amino acids according to [21]. Total amino acids in diets were analyzed using HPLC, as described by the authors of [10].

### 2.3. Laying Performance 

Mortalities were recorded daily. Egg numbers and egg weight were recorded daily. However, feed consumption was recorded weekly. Egg mass was calculated according to this formula (egg weight × egg production), while feed conversion ratio (FCR) was calculated according to grams of feed consumption/grams of egg mass produced.

### 2.4. Blood, Liver, and Ileum Sampling and Laboratory Analyses

At the end of the trial (40 weeks), 6 hens per treatment were slaughtered. The blood was collected and was centrifuged (3000× *g*) for 10 min. It was aspirated by pipette and stored in Eppendorf tubes at −70 °C. Serum concentrations of zinc (Zn), copper (Cu), triglyceride (TG), total cholesterol (CHO), glutamic oxaloacetic transaminase (GOT), and glutamic pyruvic transaminase (GPT) were determined by kits from (Nanjing Jiancheng Bioengineering Institute, Nanjing, China). The levels of serum uric acid, high-density lipoprotein cholesterol (HDL-C), and low-density lipoprotein cholesterol (LDL-C) were analyzed by commercial kits from the previous company. The HDL-C levels in serum supernatant were determined after precipitation of lipoprotein-B using phosphotungstic acid/Mg^2+^ (PTA/Mg^2+^).

Serum 3-hydroxy-3-methylglutaryl coenzyme A (HMG-CoA) reductase was determined following the manufacturer’s protocols from Sigma (St. Louis, MO, USA).

After slaughter, the liver of each hen was collected immediately, snap-frozen with liquid nitrogen, and stored at −80 °C until analysis. About 0.5 g of liver of every bird was homogenized and analyzed for CHO, TG, GOT, and GPT, as described above in the serum.

### 2.5. Oxidant and Antioxidant Status

Serum levels malondialdehyde (MDA), superoxide dismutase (SOD), total antioxidative capability (T-AOC), and copper zinc superoxide dismutase (CuZn-SOD) were analyzed as described by [22,23]. Liver tissues were homogenized in ice-cold isotonic physiological saline to form homogenates at the concentration of 0.1 g liver/mL. The samples of liver tissues were homogenized and centrifuged, and the supernatants were collected to analyze MDA, T-AOC, and SOD.

### 2.6. HSP70 mRNA Expression Assay

Total RNA was isolated from 50 mg of ileum, according to the instructions (TRIzol; Invitrogen, Carlsbad, CA, USA). The quality of RNA was examined by both native RNA electrophoresis on 1.0% agarose gel and the UV absorbance at 260 nm and 280 nm. The cDNA was synthesized from 2 lg of total RNA by a reverse transcriptase at 42 °C for 60 min with oligo dT-adaptor primer, using the protocol of the manufacturer (M-MLV; Takara, Dalian, China). The abundance of mRNA was determined based on a Step-One-Plus Real-Time PCR (ABI 7500; Applied Biosystems, Foster, CA, USA). The PCR used a kit (SYBR Premix PCR kit; Takara, Dalian, China) as described by [10]. Average gene expression relative to the endogenous control for each sample was calculated using the 2^−ΔΔCt^ method [24]. The calibrator for each studied gene was the average ^Δ^Ct value of the control group (Table 3).

### 2.7. Statistical Analyses

Data (Replicate; n = 6) were statistically analyzed by one-way ANOVA (SPSS Inc., Chicago, IL, USA). Polynomial comparisons were applied to test for linear and quadratic responses of dependent variables to dietary Thr. Inflection points in response curves at increasing dietary Thr levels were calculated following [28]. To estimate the optimal Thr requirement, a quadratic regression equation based on 95% of the maximum or minimum response was used [29,30].

## 3. Results

### 3.1. Laying Performance and Optimal Dietary Thr

The results showed that both egg mass and hen-day egg production increased quadratically (*p* < 0.05) (Table 4).

From 28 to 34 weeks, hen-day egg production presented a quadratic trend to increasing dietary Thr (R^2^ = 0.97, *p* = 0.03) at 0.58%. In addition, hen-day egg production presented a quadratic trend (R^2^ = 0.96, *p* = 0.02), at 0.58% from 28–40 weeks (Table 5).

### 3.2. Serum Biochemical Parameters

**Figure 2:**
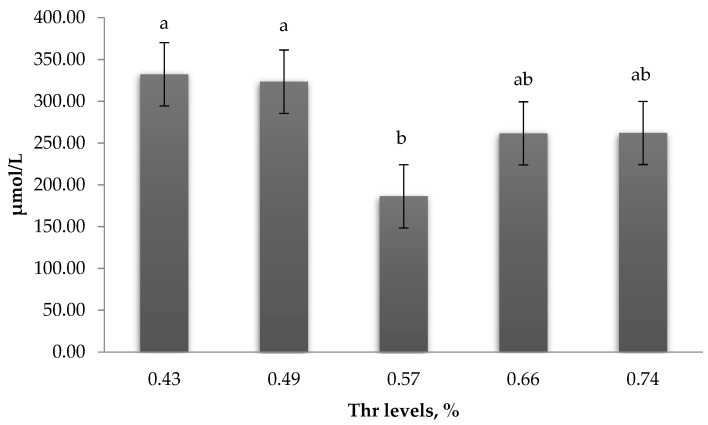
Effect of Thr levels on the levels of serum uric acid. Values are means ± standard SEM. Means on each bar with no common letter differ (*p* < 0.05).

Serum uric acid declined (*p* < 0.05) at 0.57% Thr (Figure 2) and showed a quadratic trend (R^2^ = 0.62, *p* = 0.02) at 0.59% (Table 5).

Serum total cholesterol decreased (*p* < 0.05) at 0.66% dietary Thr. Serum HMG-CoA reductase activity decreased (*p* < 0.05) at 0.49% and 0.66% dietary Thr (Table 6). No effects were observed in the liver for total CHO, HDL-C, HDL-C, and triglyceride.

Serum T-SOD increased (*p* < 0.05) at 0.49% dietary Thr. In addition, serum level of CuZn-SOD elevated (*p* < 0.05) from 0.49% to 0.66% dietary Thr (Table 7) and showed a quadratic trend (R^2^ = 0.87, *p* = 0.003) at 0.56% (Table 5).

Graded levels of dietary Thr did not affect serum or liver concentration of T-AOC, MDA, Zn, Cu, GOT, and GPT (Table 7).

### 3.3. Ileal HSP70 mRNA Expression

**Figure 3:**
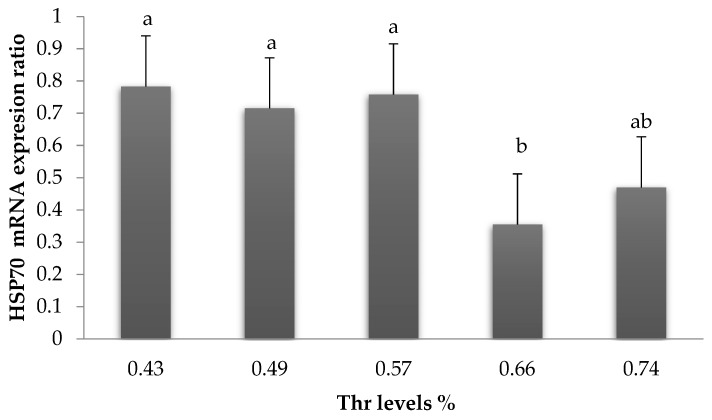
Effect of graded levels of dietary Thr on mRNA expression of ileal HSP70. Values are means ± SEM. means with different superscripts; ^a,b^ differ (*p* < 0.05).

The expression of ileal HSP70 decreased (*p* < 0.05) at 0.66% Thr (Figure 3).

## 4. Discussion

It is important to formulate accurate diets to meet the requirements of laying hens because feed ingredients are expensive. In addition, laying hens have been selected for massive egg production, resulting in greater metabolic activity and reduced thermo-tolerance [31,32]. Heat stress has adverse effects on laying hens [33,34,35]. In addition, high temperatures increase the hens’ discomfort and lead to behavioral and endocrinological changes.

In the present study, rapid panting was noticed. In addition, the expression of ileal HSP70 protein decreased (*p* < 0.05) at 0.66% Thr. It has been reported that heat shock protein protects birds from high temperatures by preventing unwanted protein aggregation and channelizing their degradation [36]. The expression of mRNA HSP70 was measured in the gut [14,37], liver [38], hypothalamus [27,39], and blood and feather [40]. Here, we focused on detecting HSP70 in ileum because it plays a vital role in digestion and absorption, as well as immunity. In addition, the effects of Thr on intestinal function were known [41,42], and the effects of heat stress on gut function become obviously clear.

Both serum CHO and serum 3-HMG-CoA reductase decreased significantly (*p* < 0.05) at 0.66% dietary Thr. It has been reported that 3-hydroxy-3-methylglutaric acid (HMG) is a potent agent for reducing serum triglyceride and cholesterol concentrations [43,44]. It has been reported that HMG causes a 40% to 50% reduction of [1–^14^C] acetate incorporation into cholesterol in male rats [45]. In addition, HMG inhibited fatty acid synthesis in vivo [46]. In vitro, HMG inhibited 3-HMG-CoA reductase [mevalonate: NADP oxidoreductase (CoA-acylating), EC 1.1.1.34] and interfered with the enzymatic steps involved in the conversion of acetate to HMG-CoA [46]. Taken together, the data suggest that dietary Thr level affect CHO, especially biochemical pathways in which HMG-CoA reductase is involved. Our findings are in agreement with the results of the previous study in broiler chickens [47]. They found that plasma CHO levels decreased significantly (*p* < 0.05) when dietary Thr was sufficient [47]. Here, we did not find a decrease in total CHO levels in the liver. Recently, it has been reported that Thr supplementation did not have an effect (*p* > 0.05) on hepatic cholesterol in Pekin ducks [48]. They suggested that dietary Thr supplementation enhanced hepatic lipid metabolism by regulating lipid synthesis, transport, and oxidation. It has been reported that there is no relationship between the plasma CHO level and the level of yolk cholesterol [49,50,51], and, consequently, liver CHO.

The levels of GOT and GPT did not change due to treatments. The enzymatic activity of GOT and GPT are indicators of liver health. These enzymes are elevated in acute hepatotoxicity, but they are decreased with prolonged intoxication [52].

In the present study, dietary Thr at 0.49% increased serum levels of T-SOD (*p* < 0.05). In addition, dietary Thr at 0.49%, 0.57%, and 0.66% increased the levels of CuZn-superoxide dismutase (Cu-ZnSOD). The present result suggests that Thr may promote the antioxidative ability of laying hens. Previous studies [22,53] also found that supplemental amino acids (L-Thr and L-Trp) increased T-SOD in serum and the liver.

Uric acid is the metabolic product of protein metabolism and has been suggested as a dominant scavenger of free radicals [54]. We found that level of serum uric acid was declined at 0.57% Thr, which confirms that sufficient Thr increases amino acid utilization. Thr is considered a limiting amino acid in low CP diets [55,56], affecting utilization of TSAA and Lys [57]. It has been reported that the levels of plasma uric acid and excreta were higher from increasing CP than from lowering CP in the diet [58]. In addition, a decrease in the level of uric acid excretion was reported with supplementing limiting amino acids, which indicated better N utilization [59,60]. The plasma urea nitrogen and uric acid have been used to estimate amino acids requirements in swine and broilers [61,62,63].

It has been reported that laying performance decreased by feeding low CP diets [64]. Here, reduction CP (14%) in the control group reduced egg production quadratically. The effect of the low crude protein diet was pronounced clearly during the late cycle of laying production (43–63 weeks of age) [65]. This study was conducted during the first cycle of egg production (28–40 weeks).

Increasing dietary Thr to 0.57% improved egg production quadratically. It has been found that egg mass and hen-day egg production were reduced (*p* < 0.05) by feeding hens a Thr deficient diet [66]. This means that increasing recognition of Thr as a critical amino acid in the diet of laying hens fed a low CP diet under high-temperature environmental climate. The current results showed that 0.58% dietary Thr based on quadratic regressions guaranteed the best egg production. The present results are in agreement with [67]. They estimated that Thr requirement was 0.57% of dietary Thr from 24 to 40 weeks in Hy-Line W36.

Egg weight, feed consumption, and FCR were similar among dietary Thr levels. Previous studies reported no effect of Thr levels on the egg weight and FCR in laying hens [68,69,70,71]. It has been indicated that total Thr deficiency beyond 0.42% decreased feed intake [72].

## 5. Conclusions

From 28 to 40 weeks of age, the optimal dietary Thr requirements to optimize egg production, serum uric acid, and serum CuZn-SOD were 0.58%, 0.59%, and 0.56%, respectively, by regression analysis. In addition, serum total cholesterol, serum HMG-CoA reductase, and expression of ileal HSP70 decreased at 0.66% Thr.

## Figures and Tables

**Table 1:** The ingredients and nutrient level of the control diet.

**Ingredients**	**%**
Yellow Corn	65.6
Soybean meal	11.0
Peanut meal (47.8%)	8.5
Soybean oil	3.0
Limestone.38% Ca	8.1
CaHPO4	1.8
L-Lys. HCL	0.23
DL-Meth	0.15
L-Thr	0.0
L-Isoleucine	0.22
L-Trp	0.04
L-Val	0.16
Premix ^1^	0.5
NaCl	0.3
Filler (Kaolin)	0.4
Total	100
**Nutrient Level ^2^**	**%**
Analyzed CP	14.0
Digestible Lys	0.69
Digestible Meth + Cysteine	0.54
Digestible Thr	0.43
Ca	3.60
P	0.43
Metabolizable Energy, Kcal/kg	2850

**^1^** Premix per kilogram of diet: Vitamins (A, 12,000 IU; E, 20 IU; D3, 2,500 IU; K3, 1.8 mg; B1, 2.0 mg; B2, 6.0 mg; B6, 3.0 mg; B12, 0.020 mg; niacin, 25 mg; pantothenic acid, 10 mg; folic acid, 1.0 mg; biotin, 50 mg). Minerals per mg: Fe, 50; Zn, 65; Mn, 65; Co, 0.250. **^2^** Values of digestible amino acids were calculated according to (Rostagno et al., 2011).

**Table 2:** Amino acids (g/kg) in experimental diets.

Amino Acids (g/kg)	Dietary Treatments (L-Thr, g/kg)				
	0.0	1.0	2.0	3.0	4.0
Digestible Thr ^1^	4.3	4.9	5.7	6.6	7.4
Arginine	8.7	8.5	8.4	8.7	8.6
Isoleucine	7.1	6.9	6.9	6.8	6.9
Lysine	7.4	7.2	7.4	7.4	7.3
Methionine	3.4	3.3	3.5	3.4	3.4
Threonine	4.5	5.2	6.0	6.9	7.8
Valine	7.7	7.5	7.4	7.6	7.5
Glycine	6.2	6.0	6.0	6.2	6.0
Serine	6.6	6.8	6.8	6.6	6.7

**^1^** Values of digestible Thr were calculated according to (Rostagno et al., 2011).

**Table 3:** Gene and primer sequence.

Gene Name	(5′–3′) Primer Sequence (5′–3′)	Reference
18s rRNA	R: ATTCCGATAACGAACGAGACT	[25]
	F: ATTCCGATAACGAACGAGACT	
GAPDH	R: TCCTAGGATACACAGAGGACCA	[26]
	F: CGGTTGCTATATCCAAACTCA	
HSP70	R: GTCAGTGCTGTGGACAAGAGTA	[27]
	F: CCTATCTCTGTTGGCTTCATCCT	

HSP70 means the 70 kilodalton heat shock proteins.

**Table 4:** Effect of graded levels of dietary Thr on laying performance of laying hens ^1,2^.

Items	Thr Levels (%)					SEM	*p*-Value		
	0.43	0.49	0.57	0.66	0.74		Thr	Linear	Quadratic
**28–34 weeks**									
Egg production, %	91.57	94.66	95.93	95.73	94.26	1.76			0.03
Egg weight, g	62.49	63.02	62.39	62.73	62.31	0.83			
Egg mass, g/hen/day	57.22	59.65	59.84	60.06	58.72	1.43			0.04
ADFI, g/hen/day	123.15	116.3	135.22	122.51	130.2	10.35			
FCR	2.16	1.95	2.26	2.04	2.22	0.18			
**35–40 weeks**									
Egg production, %	90.14	93.39	94.89	94.36	93.42	1.95			0.05
Egg weight, g	63.55	64.08	63.34	63.71	63.33	0.89			
Egg mass, g/hen/day	57.28	59.83	60.11	60.16	59.16	1.63			
ADFI, g/hen/day	117.27	115.77	122.27	115.09	121.58	9.34			
FCR	2.05	1.94	2.04	1.92	2.05	0.16			
**28–40 weeks**									
Egg production, %	90.86	94.02	95.41	95.04	93.84	1.7			0.03
Egg weight, g	63.02	63.55	62.86	63.22	62.81	0.85			
Egg mass, g/hen/day	57.25	59.74	59.98	60.12	58.94	1.43			
ADFI, g/hen/day	120.21	116.04	128.74	118.8	125.89	10.35			
FCR	2.1	1.94	2.15	1.98	2.13	0.18			

^1^ Data are means of 6 replications with 14 hens/replicate; ^2^ Throughout the entire experimental period, ^2^ birds died.

**Table 5:** Estimations of the dietary Thr requirements based on quadratic regressions.

Variables	Equations	Requirements	*p*-Value	R^2^
Hen-day egg production (28–34 week)	Y = −132.406X^2^ + 162.416X + 46.446	0.58	0.03	0.97
Hen-day egg production (28–40 week)	Y = −131.368X^2^ + 161.869X + 45.809	0.58	0.02	0.96
Serum uric acid	Y = 3207.394X^2^ − 3995.371X + 1470.705	0.59	0.02	0.62
Serum CuZn-SOD	Y = −1130.848X^2^ + 1336.98X − 1130.848	0.56	0.003	0.87

Y = Dependent variables; X = The dietary Thr level (%).

**Table 6:** Effect of graded levels of dietary Thr on the levels of lipoproteins and activities and HMG-CoA reductase of laying hens ^1,2^.

Items	Thr Levels (%)					SEM	*p*-Value		
	0.43	0.49	0.57	0.66	0.74		Thr	Linear	Quadratic
**Serum, mmol/L**									
Triglyceride	15.94	14.09	16.17	14.05	13.04	1.62			
Total cholesterol	4.67 ^a^	3.35 ^a,b^	4.26 ^a^	2.61 ^b^	3.14 ^a,b^	0.46	0.008	0.005	
High-density lipoprotein	0.32	0.25	0.25	0.27	0.18	0.06			
Low-density lipoprotein	1.01	0.88	0.9	1.0	0.84	0.14			
HMG-CoA reductase activity, ng/L	189.80 ^a^	104.41 ^b^	171.88 ^a^	103.29 ^b^	172.77 ^a^	14.97	0.0001		0.00
**Liver, μmol/gprot**									
Triglyceride	154.6	123.49	146.15	159.66	149.99	12.91			
Total cholesterol	31.51	19.68	30.1	27.64	27.59	4.63			
High-density lipoprotein	17.55	18.64	17.87	17.24	18.09	1.79			
Low-density lipoprotein	25.33	20.63	23.75	14.2	21.08	4.34			

^1^ n = 6 hens/treatment; ^2^ means with different superscripts; ^a,b^ differ (*p* < 0.05).

**Table 7:** Effect of graded levels of dietary Thr on the levels of antioxidants in the liver and serum of laying hens ^1^^,2^.

Items	Thr Levels (%)					SEM	*p*-Value		
	0.43	0.49	0.57	0.66	0.74		Thr	Linear	Quadratic
**Serum**									
T-AOC, U/mL	6.319	7.353	7.283	6.882	5.149	1.15			
T-SOD, U/ mL	131.566 ^b^	184.003 ^a^	162.439 ^a,b^	161.869 ^a,b^	178.778 ^a,b^	16.34	0.03		
CuZn-SOD, U/ mL	75.03 ^b^	103.85 ^a^	102.80 ^a^	102.34 ^a^	83.60 ^a,b^	9.03	0.008		0.001
MDA, nmol/ mL	8.071	8.93	10.043	8.945	8.795	1.01			
Zn, μmol/L	51.81	62.9	66.92	56.44	60.5	8.56			
Cu, μmol/L	18.05	17.42	18.07	17.44	16.86	0.95			
GOT, IU/L	9.45	9.84	10.22	8.6	8.92	0.65			
GPT, IU/L	3.15	2.83	3.87	3.3	3.41	0.66			
**Liver**									
T-AOC, U/mgprot	1.78	1.62	1.8	1.87	1.6	0.31			
T-SOD, U/mgprot	93.73	105.69	91.01	111.94	102.72	13.66			
MDA, nmol/mgprot	0.68	0.66	0.88	0.81	0.78	0.14			
GOT, U/gprot	40.01	41.59	42.06	38.39	39.5	3.04			
GPT, U/gprot	13.49	13.14	15.14	14.38	15.3	1.19			

^1^ n = 6 hens/treatment; ^2^ means with different superscripts; ^a,b^ differ (*p* < 0.05).
